# Thermo-Mechanical Modeling of Wire-Fed Electron Beam Additive Manufacturing

**DOI:** 10.3390/ma14040911

**Published:** 2021-02-15

**Authors:** Fatih Sikan, Priti Wanjara, Javad Gholipour, Amit Kumar, Mathieu Brochu

**Affiliations:** 1 Department of Materials Engineering, McGill University, Montreal, QC H3A 0C5, Canada; fatih.sikan@mail.mcgill.ca (F.S.); amit.kumar2@mail.mcgill.ca (A.K.); 2 National Research Council Canada, Aerospace Research Center, Montreal, QC H3T 2B2, Canada; priti.wanjara@cnrc-nrc.gc.ca (P.W.); javad.gholipourbaradari@cnrc-nrc.gc.ca (J.G.)

**Keywords:** thermo-mechanical modelling, finite element analysis, residual stresses, microstructure, Ti-6Al-4V

## Abstract

The primary objective of this research was to develop a finite element model specifically designed for electron beam additive manufacturing (EBAM) of Ti-6Al-4V to understand metallurgical and mechanical aspects of the process. Multiple single-layer and 10-layer build Ti-6Al-4V samples were fabricated to validate the simulation results and ensure the reliability of the developed model. Thin wall plates of 3 mm thickness were used as substrates. Thermocouple measurements were recorded to validate the simulated thermal cycles. Predicted and measured temperatures, residual stresses, and distortion profiles showed that the model is quite reliable. The thermal predictions of the model, when validated experimentally, gave a low average error of 3.7%. The model proved to be extremely successful for predicting the cooling rates, grain morphology, and the microstructure. The maximum deviations observed in the mechanical predictions of the model were as low as 100 MPa in residual stresses and 0.05 mm in distortion. Tensile residual stresses were observed in the deposit and the heat-affected zone, while compressive stresses were observed in the core of the substrate. The highest tensile residual stress observed in the deposit was approximately 1.0 σ_ys_ (yield strength). The highest distortion on the substrate was approximately 0.2 mm.

## 1. Introduction

In recent years, additive manufacturing (AM) technologies have revolutionized the manufacturing industry due to their original layer-by-layer processing nature and known advantages over conventional forming, forging, casting, and/or machining technologies [1]. Currently, AM is used to produce custom parts from a vast selection of materials in many industries, such as aerospace [2], automotive [3], satellite and space [3], and biomedical [4]. The main reasons for AM attracting such attention are (1) the potential of complex shape fabrication, design freedom to highlight creativity and part uniqueness [5], and (2) promising sustainability through cost effectiveness by decreasing lead time, production waste of high cost materials, energy consumption, and, thus, overall cost per part [6].

Despite several advantages, AM technology also has limitations that need to be addressed prior to becoming a mainstream production method, especially for critically loaded structures [7]. Production, geometry, and microstructural issues—such as porosity, lack of fusion between layers [8], cracking and distortion due to thermal and residual stresses [9], low spatial resolution in the final shape [10], high surface roughness [11], or need for post-processing (such as heat treatments, surface treatments, thermo-mechanical treatments or machining) [12]—hinder the advancement of AM in many fields. Such engineering challenges cause massive issues for strategic and critical industries such as aerospace where part requirements are very stringent for safety and reliability assurance. In addition, current materials used to build metal parts with AM are quite limited, and the readily available alloys for AM are inherited from welding and powder metallurgy fields. These include a few titanium [13] and aluminum [14] alloys, stainless steels [15], and nickel-based superalloys [16]. Increasing the inventory of additively manufacturable materials by developing new alloys or solving issues of current alloys are required. Thus, AM also attracts interest amongst materials and manufacturing researchers. The main efforts in AM literature are focused on several sub-groups, such as process parameter optimization to minimize defects [1], macro and microstructure control to tailor advanced properties [17], remedies for cracking problems in Ni-base superalloys [18], and developments of AM models to understand and predict thermal and mechanical phenomenon occurring during the process [19].

Electron beam additive manufacturing (EBAM) is a technique that belongs to the direct energy deposition (DED) sub-group of AM technologies [20]. EBAM involves the melting and solidification of a metal wire feedstock using an electron beam source to form the part geometry in a layer-by-layer manner. Although research on laser-based techniques has been more widely reported for AM processing of metal components, EBAM provides valuable advantages such as higher energy efficiency, faster build rate, material versatility (conductive, reflective, refractive), higher quality due to the reducing vacuum environment, and low residual stresses and part distortion [20,21]. However, in general, EBAM and DED processes are still not broadly applied and remain limited to cost-intensive parts. The reason DED often lacks cost-competitiveness against traditional production methods is due to process development complexity associated with parametric optimization and toolpath planning, which arises usually because the layer thickness during building requires corrective actions due to slight deviation in the process environment from layer to layer (e.g., heat build-up). Thus, for individual applications, the necessary trial-and-error iterative process developments become cost-/time-intensive as they involve considerable manual operator/engineer interventions. So, reliable and efficient models are of utmost importance for furthering development, and especially in the case of EBAM, they are still deficient for maturing and diversifying process applications.

Due to the complexity of AM processing, thermal and mechanical models considering heat and mass transfer are essential to calculate temperature fields to predict melt pool shape and size, cooling rates, residual stresses, and distortion. Finite element modeling is extensively used in AM processes for such purposes [21,22,23,24,25]. Some models focus on heat and mass transfer during the process and neglect mechanical phenomenon [19]. These models solve two- or three-dimensional steady-state or transient energy conservation equations with convective and radiative boundary conditions. The primary objective of such models is to monitor temperature variations within the built part in order to predict and control metallurgical aspects, such as segregation and microstructure of the material. Few of these heat transfer models also consider fluid dynamics in order to precisely calculate melt-pool dimensions and temperature distributions within the melt [26]. Such models solve conservation of mass, momentum, and energy equations and are generally computation intensive. Some other models also consider mechanical phenomena, such as yielding, thermal expansion, and elastic modulus with mechanical boundary conditions to predict residual stresses and distortion of the built geometry [27,28]. Most of these thermal and/or mechanical models neglect fluid flow for the sake of computational efficiency and focus on the prediction of residual stresses or their remedies. However, accurate calculation of the residual stresses generally requires improved features. Using appropriate material properties that consider strain rate, temperature, and phase dependence are critical for these models. In some cases, adopting hardening and creep models [24,29] reduce the error significantly [30]. However, limited material property databases force researchers to adjust their thermo-mechanical models.

This study focuses on Ti-6Al-4V, which is the workhorse alloy for the aerospace industry due to its high specific strength, high corrosion resistance, and high temperature properties [31,32]. This research targets exploring a deposition model for Ti-6Al-4V using EBAM to progress the understanding of the key factors and their effects on final deposition success, which are gauged by both reliability and performance. A thermo-mechanical finite element model (FEM) was constructed to predict residual stresses and distortion for a specified geometry, which would be purposeful for greatly reducing the amount of experimental iterations needed to optimize deposition procedures to meet the requirements (e.g., geometry, distortion tolerances) of manufactured and repaired parts. In order to validate this model, temperature variations on the substrate were recorded using thermocouples. In addition, microstructural characterization, residual stress, and distortion measurements were conducted. An important milestone in the present study was to advance the development of a part-scale thermo-mechanical model and confirm its prediction accuracy at levels similar to other reported works in the literature [21,26]. Here, it is worth mentioning that EBAM models present in the literature have mostly focused on mechanical model validation using distortion magnitudes from a singular or a few measurement points: however, in this study, residual stresses were thoroughly analyzed from nearly 100 unique measurement points. In addition, for the first time, the specific microstructural features of Ti6Al4V built by EBAM were used to validate thermal aspects of the model. In this regard, the model validation approach presented in this study gains another level of significance.

## 2. Materials and Methods

**Figure 1:**
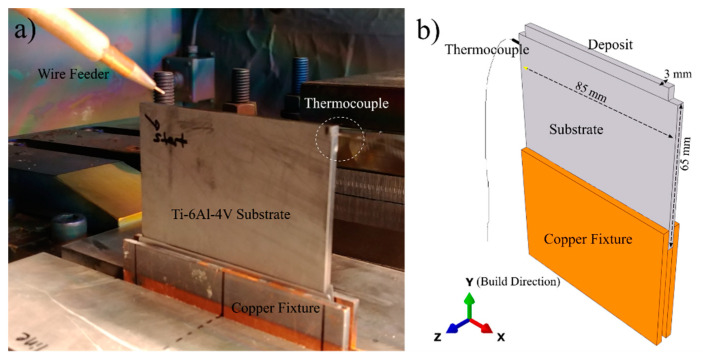
(**a**) Photograph of the experimental setup and (**b**) schematic drawing of the sample geometry.

A series of single bead thin wall structures were fabricated using a wire-fed 42 kW Sciaky EBAM and welding system (Sciaky, Chicago, IL, USA) under 5 × 10^−3^ Pa (5 × 10^−5^ mbar) vacuum at the Aerospace Manufacturing Technologies Centre of the National Research Council of Canada in Montreal, Canada to emulate additive processing. Timetal Ti64 (AMS4911M) wrought plates (Timet, Warrensville Heights, OH, USA) were used as the substrate in the deposition experiments. Substrate plates were cut with dimensions of 85 mm in width, 65 mm in height, and 3 mm in thickness. The height of the substrate was oriented parallel to the rolling direction (RD) of the plate. The top (deposition) surface of the substrate was ground with 120 grit SiC paper and cleaned with acetone prior to deposition. Temperature measurements were undertaken using a 0.5 mm diameter K-type thermocouple attached to the side of the substrate. To attach the thermocouple, a hole, approximately 1 mm in diameter, was drilled roughly 3 mm away from the top surface (Figure 1). Timetal Ti64 (AMS 4954) filler wire with a 0.9 mm diameter was used for deposition. The chemical compositions of the wire and plate are given in Table 1. In the experiment, a bi-directional scan strategy was used where subsequent layers have opposite scanning directions. Ten successive layers were deposited to produce a 5 mm build height (500 μm layer thickness). One of the experiments was stopped after depositing a single pass/layer to have a snapshot of the microstructure and residual stresses for the first layer as a point of comparison. The photograph and the schematic in Figure 1 show the substrate geometry and experimental setup. The travel speed and wire feed rate were set at 3.81 mm/s and 8.5 mm/s, respectively. Samples were cooled down to room temperature under vacuum.

A three-dimensional (3D), transient fully coupled temperature-displacement thermo-mechanical model for EBAM was developed using the commercial finite element analysis (FEA) software CAE/2018 ABAQUS™ (Dassault Systems, Detroit, MI, USA). Analysis was run on a system with 8 cores AMD Ryzen 7-1700 CPU at 3.00 GHz with 64 GB physical memory (RAM). Simulations took approximately 20 h to complete. The transient temperature fields over the part geometry in all directions were calculated from the 3D heat conduction equation, as shown in Equation (1):

(1) $$\frac{\partial }{\partial x}\left(k\frac{\partial T}{\partial x}\right)+\frac{\partial }{\partial y}\left(k\frac{\partial T}{\partial y}\right)+\frac{\partial }{\partial z}\left(k\frac{\partial T}{\partial z}\right)+Q=\frac{\partial \left({\rho C}_{p}T\right)}{\partial t}$$

where T is the temperature, k is the thermal conductivity, C_p_ is the specific heat, ρ is the density, t is the time, Q is the power generated per unit volume, and x, y, and z are local coordinates of a point on part geometry.

**Figure 2:**
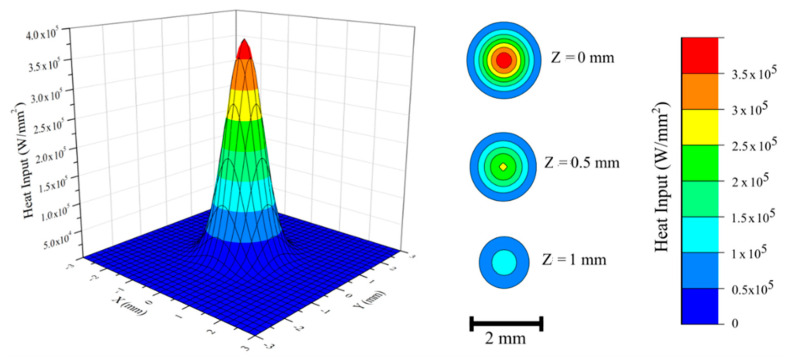
Power intensity distribution profile for an electron beam heat source model at the surface. Circular contour plots illustrate intensity variations at different penetration depths.

The high-density electron beam heat source was modeled as a conical volumetric heat source [33] with a Gaussian distribution, as illustrated in Equation (2). The intensity distribution profile was adopted and modified from the equation modeled by Rouquette et al. [34]:

(2) $$Q=\frac{2}{h}\;\left(1-\frac{z}{h}\right)\frac{3\times \eta \times V_{a}\times I_{b}}{\pi \times \emptyset_{e}^{2}}\;\exp\left(-\frac{2\left(x^{2}+y^{2}\right)}{\emptyset_{e}^{2}}\right)$$

where Q is the power generated per unit volume, h is the penetration depth, η is the heat source efficiency, V_a_ is the accelerating voltage, I_b_ is the beam current, Ø is the beam diameter, and x, y, and z are local coordinates of a point on part geometry. Heat source parameters such as beam diameter and penetration depth were determined according to the process parameters. Figure 2 shows the radial power intensity distribution profile for the heat source model at the surface. Circular contour plots on the right side of the figure illustrate the change in intensity with respect to increasing penetration depth. According to Equation (2), linear reduction in power intensity with increasing penetration depth was adopted and is shown in Figure 2. All other process parameters were the same as for the actual EBAM experiment. Heat source energy efficiency, η, was assumed to be 0.9 based on multiple literature studies [21,27,35]. In order to simulate thermal heat losses on the part, thermal boundary conditions were implemented. Forced surface convective heat losses were neglected since the EBAM process is carried out under vacuum. Radiation heat losses were considered from all outer free surfaces according to the Stefan–Boltzmann law—please see Equation (3):

(3) $$q_{\mathrm{rad}}=\varepsilon \sigma \left(T_{\mathrm{srf}}^{4}-T_{\infty }^{4}\right)$$

where ε is the emissivity, σ is the Stefan–Boltzmann constant, T_srf_ is the surface temperature of the part, and $T_{\infty }$ is the ambient temperature (298 K). Thermal parameters used in the analysis are given in Table 2. Temperature-dependent emissivity values were used. The effect of the melting/solidification on the temperature calculations was considered in the model. In order to do this, the solidus temperature, liquidus temperature, and latent heat of fusion were defined and are tabulated in Table 2. Marangoni flow (liquid mass transfer due to temperature variations and capillary forces) within the melt pool was not implemented in the model; however, to compensate this significant effect, thermal conductivity was multiplied with a correction factor of 3 for temperatures above the liquidus temperature based on the approach proposed by Lampa et al. [36] and commonly used in the literature [21].

**Figure 3:**
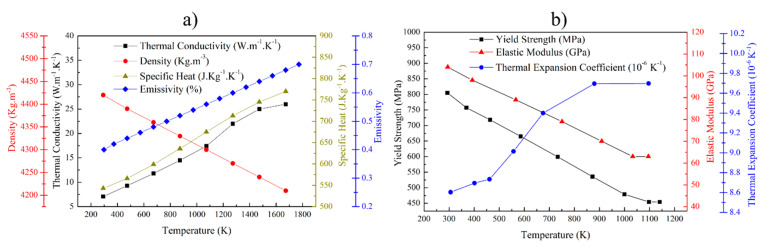
Temperature-dependent material properties used in FEA simulations: (**a**) thermal and (**b**) mechanical properties [30,37].

Figure 3 shows the temperature-dependent material properties of the Ti-6Al-4V alloy that were implemented in ABAQUS™ for both the substrate and the deposit. Thermal properties such as density, specific heat, thermal conductivity, and emissivity with respect to temperature were defined. Similarly, yield strength, elastic modulus, and thermal expansion coefficient with respect to temperature were defined as mechanical properties of Ti-6Al-4V [30,37]. For simplicity, the mechanical properties were assumed to be strain rate independent. In addition, no hardening profile was adopted in the model, i.e., perfect plasticity during deformation after the yield stress was assumed in order to reduce the computation effort and in consideration of previous high temperature tensile test studies that showed a hardening behavior which was close to perfect plasticity above 723 K for Ti-6Al-4V [38]. Anisotropy in the mechanical properties of the alloy due to microstructural texture was neglected. To simulate copper fixtures that are holding the substrate plate, mechanical boundary conditions were applied. According to the actual experimental condition, all nodes that were within a 10 mm range from the bottom of the substrate were fully fixed, meaning no displacement and rotation were allowed in any direction.

**Figure 4:**
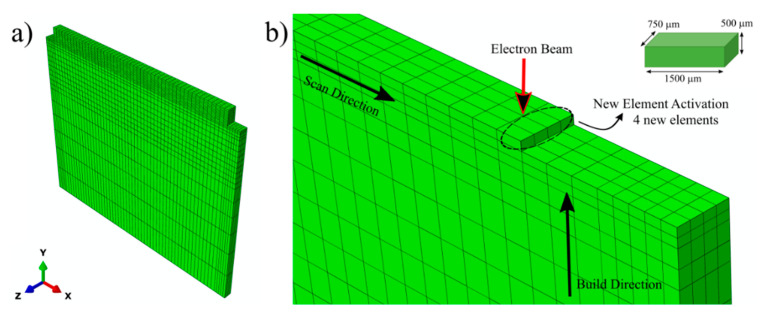
(**a**) Overview of the mesh layout for the substrate and 10-layer sample and (**b**) Mesh layout of the interface showing element activation routine and moving heat source. Inset depicts size of each elements on the deposit.

Standard solid 8-node (C3D8T) coupled displacement–temperature elements were used in the analysis. The overview of the mesh layout is shown in Figure 4a. In addition, a magnified version of the interface and the first layer’s mesh is depicted in Figure 4b. Mesh refinement was done for the elements at the interface and in the deposit to ensure accurate spatial resolution in the analysis. For substrate elements further away from the interface, a gradual coarsening in the mesh size was applied. Another mesh refinement was also applied on two side ends of the substrate to ensure the presence of a node at the thermocouple location. The mesh size on the deposit was kept constant, as shown in the inset of Figure 4b. This mesh size was selected according to the layer thickness and to ensure the presence of multiple elements within the melt pool. In order to simulate the deposition of the molten material, the “element birth technique” [21] was used. In this technique, all the elements related to the deposited material pre-exist in the model, but they only become activated with the stepwise movement of the heat source. The heat source was applied as a body heat flux on newly activated elements and previous elements within the beam diameter and penetration depth. As illustrated in Figure 4b, 4 new elements are activated in each step. Deposited elements are activated at the liquidus temperature for Ti-6Al-4V, while all other elements were set at an initial temperature, T_0_ at t = 0 s. Step time was accurately designed to imitate the process time and travel speed of the heat source in the real experiment. Step time was calculated by dividing the total length of the deposit with the multiplication of the total number of steps in 1 layer and experimental travel speed. In the simulation, the deposition of 1 layer and 10 layers takes 19.5 and 511 s, respectively. After each layer was deposited, an inter-pass waiting time of 35 s was applied to reduce heat accumulation on the part. Once full deposition is completed, another 7200 s (2 h) long step was applied for cooling to room temperature. The total deposition time was simulated as 7710 s. Finally, after the full sample geometry cools down to room temperature, the mechanical boundary conditions on the bottom of the substrate were deactivated to simulate fixture removal. A mesh sensitivity analysis was done to ensure the repeatability and reliability of the simulated results. No significant differences were observed in the residual stresses and distortion for different mesh sizes. The shown mesh size was selected for further experimental validation considering computational efficiency.

**Figure 5:**
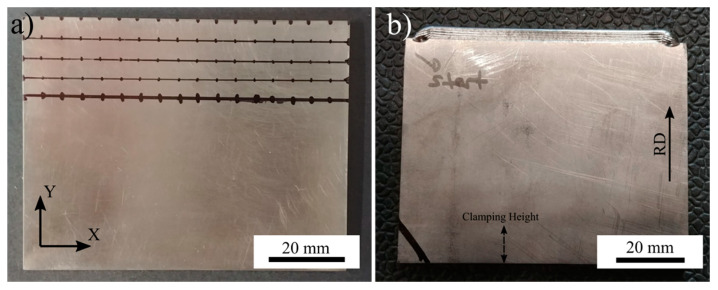
Images of the Ti-6Al-4V substrate (**a**) before and (**b**) after deposition experiment. Grid points used for XRD residual stress measurements are shown.

To validate the residual stresses on the part, experimental residual stresses for both single layer and 10-layer samples were measured with the X-ray diffraction technique (cosα method) using a Pulstec μ-X360s portable X-ray residual stress analyzer (Pulstec, Hamamatsu, Japan) with a Vanadium X-ray tube. Residual stresses of a few plates were measured before deposition to record the as-received residual stresses. As-received residual stresses were as low as ±15 MPa. Samples after deposition were not cleaned, ground, or polished before residual stress measurements to avoid the development of artificial stresses. Images of the substrate before and after deposition can be seen in Figure 5a,b, respectively. To create a measured residual stress contour map of the substrate, a region near the interface with an area of 85 × 20 mm^2^ was analyzed. This area can be seen in Figure 5a. In this area of the substrate, 5 mm apart evenly spaced grid points were demarcated with a marker pen. Residual stresses along the X axis were measured from each of these points and repeated 3 times for statistical accuracy. Measured residual stress results were used for validation of the predicted residual stress results. Distortion measurements were carried out using an ATOS Core optical 3D scanner system (GOM-Trilion Quality Systems, Seattle, WA, USA). Substrate plates were scanned before and after deposition to compare dimensional differences. Then, samples were cut roughly 20 mm away from the interface through the X-axis for investigation of the microstructure, melt pool size, and heat-affected zone (HAZ). Grinding and polishing was performed by automated techniques using a Struers grinding/polishing system. Samples were initially ground using 240 and 1200 grit SiC grinding papers; then, they were polished with a 9 µm diamond suspension on a rigid composite disk followed by a 0.02 µm silica suspension on a synthetic cloth. A few drops of H_2_O_2_ solution was added into the suspension for a better polish. Then, samples were chemically etched for microstructural examination using Kroll’s Etchant with 2 mL of HF, 10 mL of HNO_2_, and 88 mL of H_2_O. All metallographic investigation was undertaken using a Keyence VK-X laser scanning confocal/optical microscope (OM) (Keyence Canada, Mississauga, Canada).

## 3. Results and Discussion

**Figure 6:**
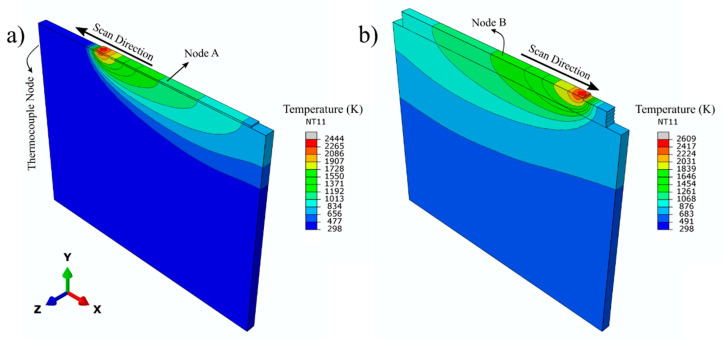
FEA predicted temperature profiles during deposition: (**a**) the first and (**b**) the last layer.

Transient thermo-mechanical simulations can yield valuable information such as temperature fields, distortion, and residual stresses as a function of process time and position. However, the reliability of such information needs to be evaluated with a series of validation experiments. Contour plots exported from ABAQUS™ in Figure 6 show the predicted temperature distributions during deposition of the first and the last layers. The thermal history of some nodes was used for validation of the model. The thermocouple node, which was selected according to the position of the real thermocouple in the experiment, is shown in Figure 6a. Similarly, nodes A and B, which are on the mid-width of the first and the last layers, are illustrated in Figure 6a,b, respectively.

**Figure 7:**
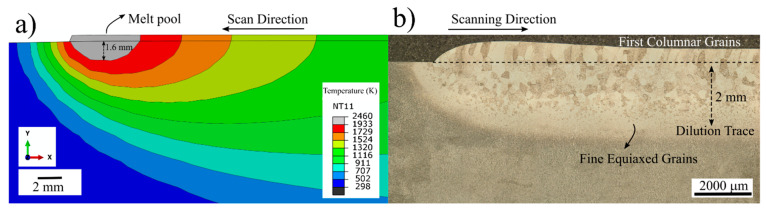
(**a**) Predicted temperature profiles showing melt pool depth during first layer in 2D and (**b**) optical microscope (OM) micrograph of the single layer sample that reveals dilution trace.

Figure 7 depicts the comparison of the simulated and experimental fusion zone (FZ). In Figure 7a, the temperature distributions during the deposition of the first layer in two dimensions (2D) are shown. Since the sample geometry is a single bead thin wall, the width of the melt pool is fixed and equals the width of the substrate plate; thus, the melt pool dimensions were only analyzed in 2D. The gray zone indicates the regions where the temperature exceeds T_liquidus_; in other words, the size of the melt pool. The depth of the gray zone was simulated to be 1.6 mm throughout the deposition of the first layer. This depth increased up to 2 mm for the last layer due to heat accumulation on the substrate. Figure 7b shows the cross-sectional OM micrograph of the single layer sample. The microstructure reveals that the melt pool depth varies between 1.5 and 2 mm for the single layer sample, which suggests good agreement with the model. Figure 7b also shows that the initially solidified β grains are relatively fine with an equiaxed morphology and have an approximate grain size of 67 ± 13 μm. On the other hand, the microstructure of the FZ shows gradual coarsening with further distance from the fusion interface toward columnar grains parallel to the build direction in the deposit microstructure. Detailed analysis of the microstructure in the FZ, HAZ, and calculated cooling rates will be discussed later in this paper.

**Figure 8:**
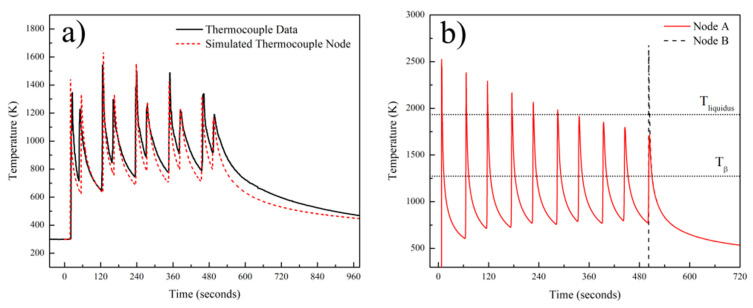
(**a**) Plot showing comparison between predicted and experimentally measured temperature profiles and (**b**) predicted thermal history of the node A and node B.

For node A and B (as identified in Figure 6), the temperature sequences were experimentally recorded and simulated as a function of processing time. Figure 8a compares the predicted results from the thermocouple node in the FEM model against the measured temperature data from actual experimentation. This figure shows that the predicted and experimental temperature profiles are in good agreement. The temperature profile plots gathered from nodes A and B were used to comment on the thermal history of the sample and to calculate the local cooling rates for the first and last layer of the deposit. Figure 8b depicts the thermal history of nodes A and B along with the key temperature thresholds of significant microstructural changes (T_liquidus_, 1933 K and T_β-transus_, 1273 K) for Ti-6Al-4V alloy. Each temperature peak in Figure 8 corresponds to a layer pass. After each peak, a cooling period can be seen. This period corresponds to the summation of cooling time due to the heat source moving away from the node of interest and inter-pass waiting time. Comparison of the predicted and experimental temperature peaks for the first few layers showed that the model does an over-estimation of approximately 100 K. Further analysis revealed that the mismatch is the highest for the first 3 layers. The calculated mismatches in the peak temperatures and the corresponding layer number can be listed as 6.53% for the 1st layer, 7.92% for the 2nd layer, and 5.57% for the 3rd layer. The average mismatch for the rest of the layers was 2.3%. The agreement indicates that the computed temperature fields can be used for microstructural predictions and the residual stress calculations. From the thermal history of the sample shown in Figure 8b, it is possible to state that every point in the first layer melts and solidifies with each pass at least 5 more times after deposition. Considering the position of the node A, the melt pool depth, and the layer thickness, this finding is plausible. It should be noted that the melt pool depth (2 mm) is at least 4 times the layer thickness (500 μm ascertained as explained below). Another important feature of the thermal history is the number of events where the local temperature exceeds T_β-transus_, the allotropic transformation. According to Figure 8b, it is clear that the temperature of the first layer exceeds T_β-transus_ for each layer throughout the deposition process for 10 layers. This means that every local point in the deposit experiences an α to β phase transformation during each heating cycle and a β to α phase transformation during each cooling cycle. This phenomenon is expected to cause homogenization of the microstructure in the deposit. The cooling rates were calculated by taking the first derivatives of the plots in Figure 8b. Calculated cooling rates for the first layer at node A can be listed as: 264 K/s at T_liquidus_ (during first solidification), 89 K/s at T_β-transus_ (during first pass), 52 K/s at T_β-transus_ (during last pass). Similarly, the cooling rate of the last layer at node B was calculated as 49 K/s at T_β-transus_. This result indicates that the final cooling rate at T_β-transus_ is quite similar everywhere over the build height in the deposit and approximately equal to 50 K/s.

**Figure 9:**
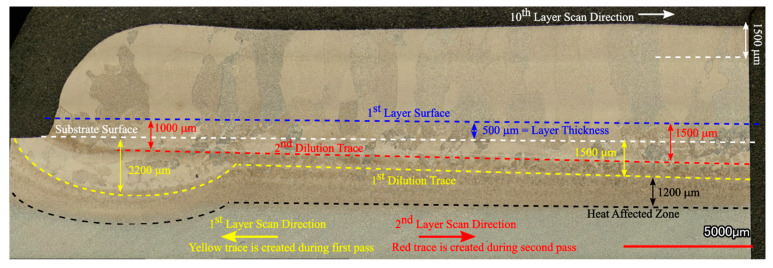
Low magnification OM micrograph of the 10-layer Ti-6Al-4V sample showing macrostructure overview of the heat-affected zone (HAZ), fusion zone (FZ) and the deposit.

**Figure 10:**
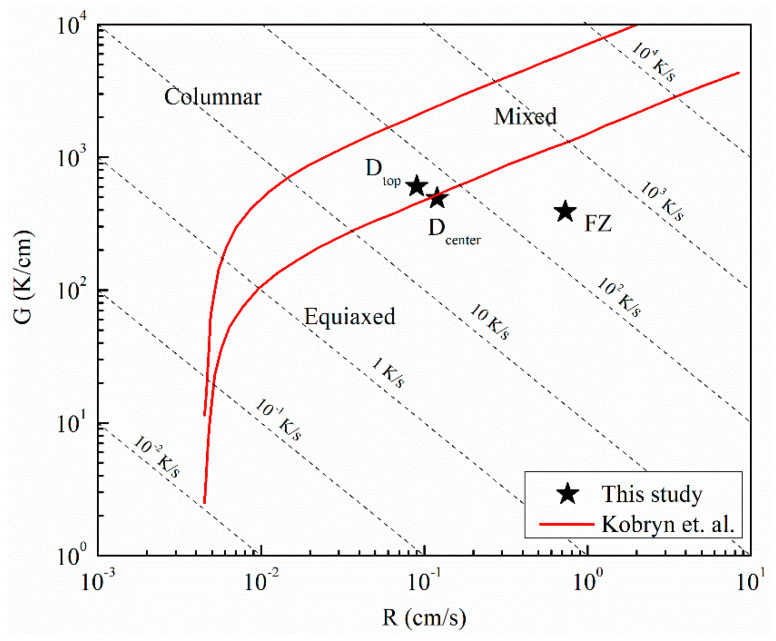
The Ti-6Al-4V solidification map [45] showing the calculated G and R values from various regions of the sample: D_top_ (top of the deposit), D_center_ (mid-height of the deposit) and FZ (fusion zone).

Figure 9 shows the OM micrograph of the 10-layer sample macrostructure that indicates a wider HAZ (extending approximately 1.2 mm) compared to the single layer sample. The main reason for this phenomenon is the longer exposure of the substrate to the elevated temperatures during the process. The temperature values recorded by the thermocouple in Figure 8a can provide an idea of the thermal cycle experienced in the HAZ. It can be stated that the temperatures in that zone varied between 800 and 1550 K for most of the deposition time. This resulted in the coarsening of the α laths in the rolled plate. It has been reported that the HAZ is observed when the temperature exceeds 708 °C, where α dissolution starts to affect microstructure [39]. Figure 9 gives a detailed description of the thermal events and their corresponding effects on the microstructure of the sample. The region shown in the figure is the end-side of the odd numbered layers. The yellow trace and arrow illustrated in the figure reveal the depth of the melt pool in the first layer. Similar to the single layer results, the melt pool depth is approximately 1.5 mm. Note that a wide curved melt pool trace is visible at the end of the scan paths. In addition, the melt pool depths appear to be slightly shorter at the beginning of the scan paths. These features were created due to the acceleration and deceleration routines of the EBAM machine during processing and do not reflect the stable moving melt pool dimensions. After deposition of the first layer, the second layer was started with an opposite scanning direction, as shown with the red arrow. During the deposition of the second layer, another dilution trace was created, which is shown with the red dashed line. The second dilution trace can also be used to validate the melt pool depth. The height of the first layer was marked on the micrograph, 500 μm above the substrate level. The distance between the first layer surface and 2nd dilution trace was measured as 1.5 mm, revealing the melt pool depth once again. The macrostructure of the 10-layer sample showed equiaxed prior β grains at the FZ of the substrate and columnar prior β grains parallel to the build direction at the deposit. This type of grain structure is common in the literature and formed due to the directional heat flux during cooling [40,41]. Cooling in the FZ of the substrate occurs faster and in multiple directions since the substrate acts as a heat sink [42]. This results in relatively finer irregular or equiaxed prior β grains [43]. However, cooling in the deposit is strongly directional, and the heat flux is opposite to the build direction. Then, the columnar grains grow parallel to the build direction along the maximum temperature gradient with epitaxial growth [44]. In order to reinforce these discussions, thermal gradients, G, and solidification rates, R, from various regions of the sample were calculated using the thermo-mechanical model. Calculated G and R values were compared with solidification maps of Ti-6Al-4V from the literature [45] to predict solidification microstructures. Figure 10 shows the Ti-6Al-4V solidification map along with the calculated G and R values from these regions. The plot shows that calculated G and R from the FZ is in the equiaxed grain morphology map field. This result agrees well with the microstructural observations in Figure 9. On the other hand, calculated G and R values from the top and mid-height of the deposit are located at the mixed grain morphology map field. Although the majority of the grains in these regions are in columnar nature, there are few grains with equiaxed morphology as well. It is safe to report that equiaxed grains could still form under these solidification conditions. The final note from the solidification map is the difference between predicted cooling rates from the FZ and the deposit. The higher predicted cooling rate in the FZ explains the finer grain structure of the corresponding region shown in Figure 9. Another structural feature of the Ti-6Al-4V wire-feed deposits in the literature are the layer bands observed in the microstructure. These bands are shown to be in curved shapes for multi-bead deposits [19,40] or straight lines for single-bead deposits [42,46]. These bands are known to originate from α/β allotropic phase transformations during multiple heating and cooling cycles [19,40]. Brandl et al. stated that the zones that experience temperatures above T_β-transus_ during the deposition of the last layer become free of this band structure [40]. This band-free region is called the “transient region” in the literature [47] because of its incomplete thermal history. On the other hand, zones that are no longer experiencing temperatures above T_β-transus_ are called the “steady-state region”, since their thermal history is completed and is no longer undergoing phase transformations. In this study, the microstructure of the 10-layer sample illustrated in Figure 9 shows no evidence of a band structure. Considering the predicted thermal history of the sample shown in Figure 8a, the whole sample was heated above T_β-transus_ even during the last pass. This explains the lack of bands in the microstructure for the 10-layer sample. It can be stated that the 10-layer sample is fully in the “transient region”, and more layers are required to observe the band structure for this alloy under these processing conditions. Finally, the dilution trace of the last layer can be observed in the microstructure approximately 1.5 mm away from the top of the sample. Dilution traces for the intermediate layers were not observed probably due to the homogenization of the microstructure during the process. The last trace is visible since the sample was never heated to homogenize the microstructure after that layer.

**Figure 11:**
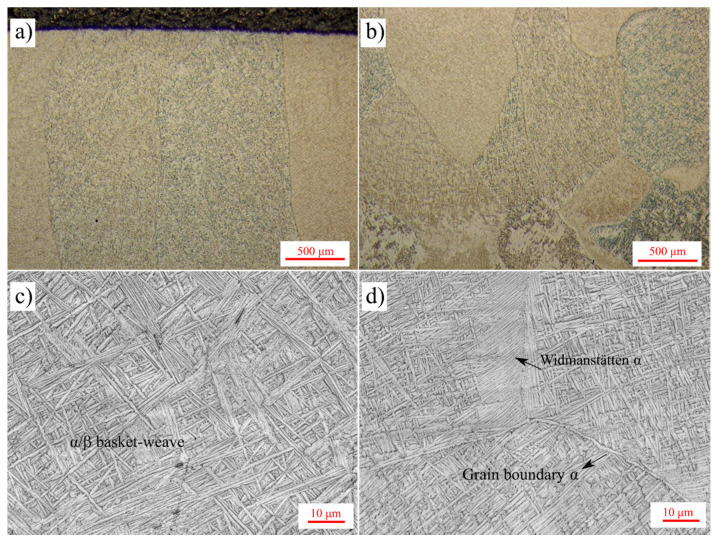
Higher magnification OM micrographs of the 10-layer Ti-6Al-4V sample from (**a**,**c**) first layers and (**b**,**d**) last layers of the deposit.

**Figure 12:**
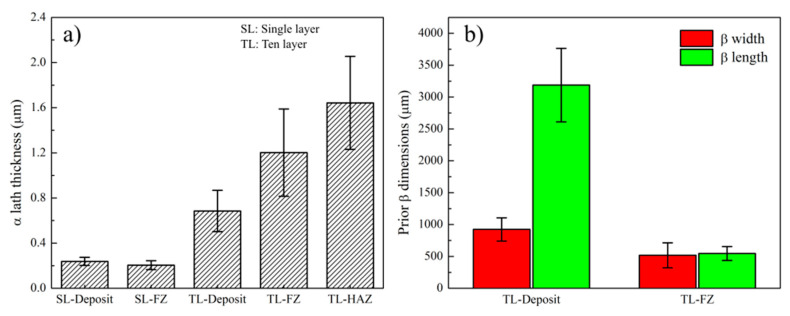
Plots showing dimensions of microstructural features such as (**a**) α lath thickness and (**b**) prior β dimensions for the single layer and 10-layer Ti-6Al-4V samples.

Higher magnification OM micrographs of the 10-layer Ti-6Al-4V sample are shown in Figure 11. All micrographs were taken from the deposit side, while Figure 11a,c are showing the upper portion and Figure 11b,d are showing the lower portion of the sample near the FZ. A basket-weave α/β microstructure was observed within the prior β grains as seen in Figure 11c,d. A small amount of allotriomorphic (grain boundary) α or Widmanstätten α phase was observed in the lower side of the sample. It is important to recall that the calculated cooling rates (52–49 K/s) were below the critical cooling rate (410 K/s) for α’ martensite formation [48]. Observation of the basket-weave α/β microstructure further validates the predicted thermal history. In addition to the micrographs, the size of the microstructural features from various zones of the single layer and ten-layer samples are reported in Figure 12. Prior β grain widths were measured as 923 ± 183 μm and 517 ± 196 μm for the deposit zone and FZ, respectively. Similarly, prior β grain lengths were measured as 3.2 ± 0.6 mm and 0.6 ± 0.1 mm for the deposit zone and FZ, respectively. The average length of the columnar prior β grains in the deposit extend up to 6-7 layers, further highlighting the epitaxial growth of β grains over subsequent layers. On the other hand, equiaxed prior β grains within the FZ had an aspect ratio close to one, as shown in Figure 12b. Another key microstructural feature for Ti-6Al-4V alloy are the needle-shaped α laths. During cooling through T_β-transus_, the high-temperature body centered cubic (BCC)-β phase undergoes allotropic phase transformation to the hexagonal closed packed (HCP)-α phase. This HCP-α phase begins to nucleate at the β grain boundaries according to the crystallographic relationship known as Burgers Orientation Relationship (BOR). This relationship dictates that from the —{110} _β_ || (0001) _α_, <11$\bar{2}$0> α || <1$\bar{1}\bar{1}$> _β_ –, 12 possible α variants can nucleate at the boundary of the β grains. Specifically, {110} planes of the BCC-β phase set the basal planes of the HCP-α phase [32,43,49,50,51]. Once the α laths nucleate, they grow and form a basket-weave microstructure. The size of the individual α laths also reflect the effect of thermal history and the cooling rate on the final microstructure [31,52]. In the observed microstructure, no distinct change in α lath thickness was observed throughout the 10-layer deposit. This is also illustrated in Figure 11c,d. This is an expected result considering the predicted thermal history, since all layers undergo a final allotropic phase transformation and cool down with a similar cooling rate after the last pass. However, the measured α lath thicknesses were coarser in the FZ and HAZ of the substrate. Measured α lath thicknesses were 0.7 ± 0.2 μm, 1.2 ± 0.4 μm, and 1.6 ± 0.4 μm for the deposit, FZ, and HAZ, respectively. The reason for the coarser microstructure in the FZ and HAZ is that at some point, they stop experiencing allotropic phase transformations as new layers are added, due to the increasing distance from the heat source. However, the microstructure is still exposed to sub-transus temperatures due to the heat diffusion. Thus, a gradual coarsening occurs with every new layer. The measured α lath thicknesses for the single layer sample are 0.24 ± 0.04 μm and 0.21 ± 0.04 μm from the deposit and FZ, respectively. Laths were approximately 0.4 μm thinner in the single layer sample compared to the ten-layer sample. This can be explained with the difference between the final cooling rates of the single layer sample (89 K/s) and the ten-layer sample (49 K/s), as the simulations predicted. In the literature, the dimensions of the microstructural features for DED fabricated Ti-6Al-4V parts depend on the build height and heat source types [53]. For example, the length of the prior β grains reported in the literature for 10 mm long samples are 5.1 ± 3.2 mm and 1.2 ± 0.6 mm for the electron beam and laser, respectively [53]. The prior β lengths in this study are slightly shorter, but this can be due to the smaller sample height. Further layers would cause longer prior β grains. On the other hand, α lath thickness for electron beam, laser, and tungsten inert gas torch (TIG) DED deposits usually vary between 0.5 and 2 μm [42,49,53,54,55]. Although general understanding in the literature suggests that increasing the cooling rate results in a reduction in α lath thickness [40,48,50], this decrease does not occur linearly with the cooling rate change [31]. Lütjering stated that the α lath thickness decreased drastically from 5 to 0.5 μm due to the changes in cooling rate in the range of 0.02 to 2 K/s. A further increase in the cooling rate up to 150 K/s resulted in only a 0.3 μm decrease [31]. In another study, Kelly studied the effect of cooling rate on the α laths thickness and showed micrographs having α laths with approximately 1, 0.7, and 0.3 μm thickness for 0.6, 10, and 94 K/s, respectively [47]. Therefore, it can be concluded that the calculated cooling rates and the corresponding microstructural features agree well with the previous literature studies with similar cooling rates.

**Figure 13:**
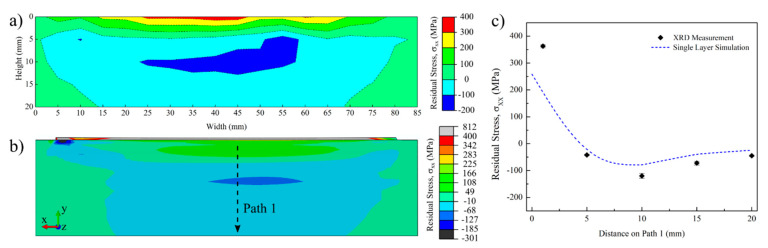
Contour plots showing the residual stresses along the scan direction (XX) for the single layer Ti-6Al-4V sample and substrate: (**a**) measured by XRD, (**b**) predicted from the FEM and (**c**) compared along Path 1.

Residual stresses in AM processes mainly develop because of the thermal cycles experienced with the addition of new layers. Local differences in temperatures and their corresponding non-uniform volume expansions and contractions result in their development [1,21]. Figure 13 shows the measured and predicted residual stresses along the scan direction (x) for the single layer sample. Residual stresses were compared along the scan direction (x) since they were the highest and dominant residual stress on the substrate. Residual stresses beyond 20 mm distance from the deposit/substrate interface are not shown since they were relatively low (0–10 MPa) and considered negligible. Experimental residual stresses developed in the substrate shown in Figure 13a were measured using XRD from each of the pen marks depicted in Figure 5a. Corresponding predicted residual stresses are given in Figure 13b with the exported image from ABAQUS™. A residual stress color scale was adjusted to be similar to the one in Figure 13a for visual comparison. Residual stresses above 400 MPa, as shown in the gray zone, were only calculated on the deposit. Thus, the following observations and discussions can be derived from the results of the single layer sample. First, predicted and measured residual stress contour plots are in good agreement. The residual stresses are highest along the scan direction, concurring with previously reported findings in the literature for AM parts [1,56]. The deposit and HAZ on the substrate are under tension, while the core of the substrate is under compression for the single-layer sample. The highest tensile residual stress on the deposit and the substrate are approximately 800 (1.0σ_ys_) and 400 (0.5σ_ys_) MPa, respectively. Similarly, in the literature, the ratio of the maximum residual stress to the alloy yield strength at room temperature for Ti-6Al-4V deposits were reported to be around 1.0 σ_ys_ [25]. The results also showed that the depth of the tensile residual stress region is approximately 4 mm from the substrate surface, which is similar to the HAZ depth. Due to the heating, during the new layer deposition, the deposited material and HAZ expand as its yield strength decreases. However, this expansion is hindered by the cooler substrate material. Thus, compressive stress is observed on the substrate material. Due to subsequent cooling, the deposited material and HAZ try to contract. Contraction is again obstructed by the surrounding material. Although contraction is easier initially due to the lower yield strength at elevated temperatures, it becomes harder as the part cools down. This results in a tensile stress in the deposited material and HAZ [21]. Figure 13c shows the measured and predicted residual stresses along Path 1, as shown in Figure 13b. The predicted residual stress profile has a decent fit with the experimental data points. The highest error was observed to be at the deposit/substrate interface, near x = 0 along Path 1. The measured residual stress at this point is approximately 100 MPa higher. One possible reason for this error could be the overflow of the deposited material to the substrate in the experiment, which was not simulated in the model.

**Figure 14:**
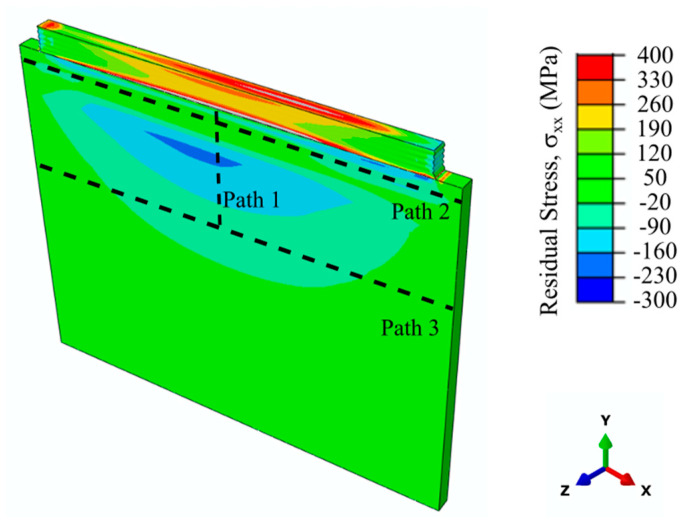
Predicted residual stresses along the scan direction for 10-layer Ti-6Al-4V deposit and substrate. Figure also illustrates pre-defined paths for residual stress plots.

The predicted residual stresses along the scan direction (x) for the 10-layer Ti-6Al-4V sample and pre-defined three paths are shown in Figure 14. A residual stress profile similar to the single layer sample was calculated for the 10-layer sample. A compressive stress region is located at the core of the substrate just below the HAZ, while most of the deposit is under tension. Tensile residual stresses were calculated to be higher at the interface or at the top and mid-width of the deposit. The highest calculated tensile residual stress was approximately 400 MPa in the deposit. Predicted residual stresses were considerably lower for the 10-layer sample compared to the single layer sample. The major reason of this phenomenon is the stress relaxation and softening in the 10-layer sample due to subsequent heating cycles and longer heat exposure. Another affecting factor for such phenomenon can be that the single-layer sample was deposited on the cold substrate at the room temperature, while the last layer of the 10-layer sample was deposited on a surface, which was already relatively hot. This could work as a pre-heat treatment before processing [57]. It was previously reported in the literature that the elevated temperatures and reduced temperature gradients experienced during EBAM processing are the main basis of the low residual stresses on the substrates [49]. The second affecting factor that could influence the residual stresses are the solid-state phase transformations occurring due to the multiple thermal cycles [30,58]. Denlinger et al. stated that volumetric changes due to allotropic phase transformations cause transformation strains within the alloy. These strains could potentially oppose contraction strains during cooling and relieve residual stresses [27,30]. Similarly, Elmer et al. studied the lattice expansions of Ti-6Al-4V during allotropic transformations via in situ high-energy synchrotron X-ray radiation and found that α and β phases have dramatically different changes in their lattice parameters during allotropic transformation. These differences in lattice parameters and thermal expansion behaviors are considered to be the origin of the stress relief in Ti-6Al-4V during allotropic transformations [58]. The residual stress profiles encountered in this study for the thin wall substrate were found to be similar to the ones in the literature for the bulky substrates [25]. Mukherjee et al. showed the evolutions of the residual stresses progressively in each layer. Their study revealed a compressive stress region on the substrate below the HAZ and a tensile stress region on the deposit. This tensile region was most pronounced at the mid-width of the deposit. Each layer deposition resulted in the tensile region shifting to an upper layer on the deposit. Residual stresses on the previous layers were stress relieved partially because of the heating during the process [25]. These findings are in good agreement with the residual stress evolution observed from the single layer to the 10-layer sample of this study. Figure 14 shows a gradual increase in the predicted tensile residual stresses with each layer, further highlighting the shift in residual stresses with each pass. Residual stresses in the 10-layer sample were further compared with the experimental measurements using the pre-defined paths shown in Figure 14. Path 1 extends 20 mm from the deposit/substrate interface to the substrate at the mid-width of the substrate. Path 2 and Path 3 are located approximately 1 mm and 20 mm away from the interface, respectively.

**Figure 15:**
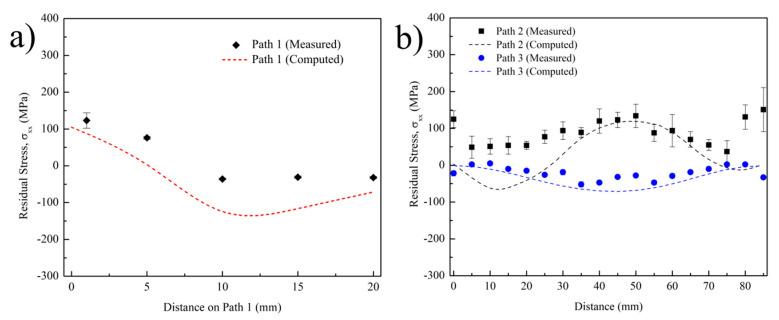
Predicted and measured (XRD) residual stress comparison plots along scan direction on (**a**) Path 1 and (**b**) Paths 2 and 3 for 10-layer Ti-6Al-4V substrate.

Calculated and measured residual stresses along the scan direction on Path 1 and Paths 2 and 3 are shown in Figure 15a,b, respectively. Both figures show that acceptable agreement was achieved between the residual stress predictions and experimental values. Calculated and measured profiles fit well with each other for the majority of the path lengths. The highest error was observed on Path 3 near the side ends of the plate. This might be due to the fact that the experimental deposit is slightly longer than the simulated geometry. In addition, metal overflow at ends is more effective because of the acceleration/deceleration routine of the EBAM machine, as previously mentioned. Calculation error and residual stresses were low on Path 2, as it was relatively far from the effective thermal gradient and high residual stress region of the substrate. Figure 15a better illustrates the residual stress variations along the substrate height. Residual stresses on the substrate decrease from approximately 125 MPa down to −30 MPa with increasing distance from the deposition interface. When compared with the single layer sample, it is clear that residual stresses below the HAZ were relieved from approximately −100 to −30 MPa. This is probably the result of stress relieving on the substrate due to elevated temperatures experienced with each layer deposition. Although calculation and measurement follow a similar trend with increasing distance along Path 1, the predicted values are approximately 50 MPa toward compressive. The possible reason of this discrepancy between simulation and the experiment could be the defined material properties in the model. Lu et al. studied the effect of different material properties on the calculated residual stresses for Ti-6Al-4V and found that small differences in yield strength, coefficient of thermal expansion, and elastic modulus could significantly change the final residual stress profiles [59]. Considering that these properties depend on the microstructure/texture that are constantly changing with the temperature during AM, they are locally different even in a single part. So, it is relatively hard to predict the exact properties at all times.

**Figure 16:**
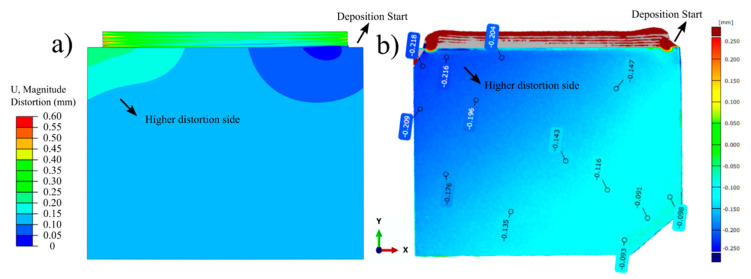
Contour plots showing (**a**) predicted and (**b**) measured out-of-plane distortion maps of the 10-layer Ti-6Al-4V deposit and the substrate.

Due to the thermal gradients and uneven thermal expansion throughout the part, the initial geometry is continuously experiencing distortion during the process. Figure 16 depicts the contour plots showing the comparison of predicted and measured out-of-plane distortion maps of the 10-layer Ti-6Al-4V deposit and the substrate. Figure 16a shows that the calculated distortion is relatively low, and the magnitude varies between 50 and 250 μm. The highest distortion is localized at the end side of the first layer pass. The measured out-of-plane distortion map is in good agreement with the predicted values, as shown in Figure 16b. Similar to the model predictions, the highest distortion was measured on the end side of the first layer pass. In addition, the deposit region is shown to have high distortion as an experimental artifact. During the measurement procedure via the ATOS Core Optical 3D scanner, the geometry was scanned before and after deposition. Since the deposit was not present in the initial scan (before deposition), the contour map shows a high distortion region on the top. Measured out-of-plane distortion magnitudes also vary between 90 and 200 μm. Measured values are shown to have negative magnitude; this reveals that the substrate plane is distorted inwards, meaning the plate is thinner after deposition. Such deformation is plausible, since the highest residual stresses are tensile along the scan direction (x) and are experienced at the top of the substrate. In our previous study, highest substrate distortion was observed at the same corner with the magnitude of approximately 300–400 μm for 50 mm build height under the same conditions [49]. In this study, similar or slightly lower distortion was observed for a 5 mm build height. Previous studies in the literature showed that the substrate distortion evolution rate during DED processing is much more pronounced during the initial few layer deposition and gradually diminishes throughout the process for Ti-6Al-4V components [27,59]. A simple explanation for this is the shifting of high thermal gradients and residual stresses away from the deposit with increasing build height. In addition, the decrease in experienced cooling rates would lower the distortion rate. These may reasonably account for the similar distortion observed for two different build heights.

In future work, the developed model will be utilized to investigate EBAM process planning as well as parametric optimization for both manufacturing and repair methodology processing in which the substrate/part integrity is also critical for return to service. Acquired knowledge from the model will be used to facilitate process development to assure suitable heat dissipation and minimize distortion for complex part geometries, such as fan blades that have twisted double curvature surface profiles.

## 4. Summary and Conclusions

In this study, a 3D transient fully coupled thermo-mechanical model for EBAM was developed using the commercial FEA software ABAQUS™ in order to investigate and plan Ti-6Al-4V deposition methodology and process. The developed model was calibrated for computational efficiency through a series of mesh sensitivity studies. The developed model was also validated using multiple EBAM and characterization experiments. Thermal validation through melt-pool size analysis, thermal history analysis, and microstructure analysis showed that the developed model is extremely reliable to predict local temperatures and cooling rates, melt-pool and dilution depths, and the resulting macro and microstructure over the deposit and the substrate. The microstructure of the EBAM deposit was observed as an α/β basket-weave structure. Initial β grains within the FZ and first layers were observed to have equiaxed morphology. On the other hand, β grains in the lateral layers of the deposit were observed to have mostly columnar morphology parallel to the build direction with few exceptions. Predicted G and R values were validated using solidification maps [45] and microstructure. No banding in the structure was observed due to the low build height and thin wall geometry of the samples. Mechanical validation was undertaken via comprehensive residual stress and distortion measurements. Predicted and measured residual stresses were in good agreement, further highlighting the reliability of the developed model. Residual stresses were more pronounced along the scan direction. Tensile residual stresses were dominant over the deposit and HAZ, while compressive residual stresses were observed at the core of the substrate. Higher residual stresses along the substrate were observed for the single layer sample compared to the 10-layer sample. Increasing the build height and thus the longer exposure to elevated temperatures are thought to be the origin of this stress relaxation on the substrate. ATOS Core 3D optical measurements revealed that the model predictions were very accurate on calculating the distortion profile and magnitude on the substrate. The highest distortion observed on the substrate was approximately 250 μm, revealing success on maintaining initial geometry.

## Figures and Tables

**Table 1:** Chemical composition (wt.%) of Timetal Ti64 wire and substrate used in the study.

Component	Al	V	Fe	C	O	N	H	Y	Ti
AMS 4911M Plate	6.21	4.00	0.18	0.006	0.18	0.005	-	0.005	Balance
AMS 4954 Wire	6.66	4.18	-	0.03	0.18	0.007	0.003	0.005	Balance

**Table 2:** Parameters used in FEA-electron beam additive manufacturing (EBAM) simulation.

**Heat Source Efficiency, η**	0.9 [35]	$\mathrm{Ambient}\;\mathrm{Temperature},\;T_{\infty }$	298 K
**Initial Temperature, T_0_**	298 K	**Liquidus Temperature, T_liquidus_**	1933 K [19]
**Inter-pass Waiting Time**	35 s	**Stefan–Boltzmann Constant, σ**	5.67 × 10^−8^ W/m^2^K^4^
**Solidus Temperature**	1877 K [19]	**Latent Heat of Fusion**	360 kJ/kg [19]

## Data Availability

The authors confirm that the data supporting the findings of this study are available within the article.
